# Design and development of a new ambr250® bioreactor vessel for improved cell and gene therapy applications

**DOI:** 10.1007/s10529-021-03076-3

**Published:** 2021-02-02

**Authors:** Marco Rotondi, Ned Grace, John Betts, Neil Bargh, Elena Costariol, Barney Zoro, Christopher J. Hewitt, Alvin W. Nienow, Qasim A. Rafiq

**Affiliations:** 1 Advanced Centre for Biochemical Engineering, Department of Biochemical Engineering, University College London, Gower Street, London, WC1E 6BT UK; 2 Sartorius Stedim Biotech, York Way, Royston, SG8 5WY UK; 3 Aston Medical Research Institute, School of Life and Health Sciences, Aston University, Birmingham, B4 7ET UK; 4 School of Chemical Engineering, University of Birmingham, Edgbaston, Birmingham, B15 2TT UK

**Keywords:** Ambr250, Bioreactor, Vessel, Bioprocessing, Automation, hMSC, T-cell

## Abstract

The emergence of cell and gene therapies has generated significant interest in their clinical and commercial potential. However, these therapies are prohibitively expensive to manufacture and can require extensive time for development due to our limited process knowledge and understanding. The automated ambr250® stirred-tank bioreactor platform provides an effective platform for high-throughput process development. However, the original dual pitched-blade 20 mm impeller and baffles proved sub-optimal for cell therapy candidates that require suspension of microcarriers (e.g. for the culture of adherent human mesenchymal stem cells) or other particles such as activating Dynabeads® (e.g. for the culture of human T-cells). We demonstrate the development of a new ambr250® stirred-tank bioreactor vessel which has been designed specifically to improve the suspension of microcarriers/beads and thereby improve the culture of such cellular systems. The new design is unbaffled and has a single, larger elephant ear impeller. We undertook a range of engineering and physical characterizations to determine which vessel and impeller configuration would be most suitable for suspension based on the minimum agitation speed (N_JS_) and associated specific power input (P/V)_JS_. A vessel (diameter, T, = 60 mm) without baffles and incorporating a single elephant ear impeller (diameter 30 mm and 45° pitch-blade angle) was selected as it had the lowest (P/V)_JS_ and therefore potentially, based on Kolmogorov concepts, was the most flexible system. These experimentally-based conclusions were further validated firstly with computational fluid dynamic (CFD) simulations and secondly experimental studies involving the culture of both T-cells with Dynabeads® and hMSCs on microcarriers. The new ambr250® stirred-tank bioreactor successfully supported the culture of both cell types, with the T-cell culture demonstrating significant improvements compared to the original ambr250® and the hMSC-microcarrier culture gave significantly higher yields compared with spinner flask cultures. The new ambr250® bioreactor vessel design is an effective process development tool for cell and gene therapy candidates and potentially for autologous manufacture too.

## Introduction

Cell and gene therapies (CGTs), such as human mesenchymal stem cells (hMSCs; Silva Couto et al. 2020) and CAR-T cells (Wang and Rivière 2016), present a novel therapeutic modality to treat a range of chronic, age-related conditions and address current unmet clinical need. Despite their clinical promise, however, currently approved CGTs suffer from a lack of scalable manufacture, high costs (> $150,000 per dose), poorly defined manufacturing processes and a lack of effective small-scale models to support process development activity (Vormittag et al. 2018). We have previously demonstrated the adaptation and amenability of the automated ambr15® microbioreactor system for high-throughput process development of hMSCs (Rafiq et al. 2017). This follow physical characterisation studies of the ambr®15 which demonstrated comparability with an industrial Chinese Hamster Ovary (CHO) cell line (Nienow et al. 2013). However, for autologous cell therapy manufacture, for example with CAR-T therapies, a larger scale bioreactor will be required to generate the cell numbers required, most likely in the 100–250 ml range (Vormittag et al. 2018). Moreover, for hMSC and other allogeneic cell therapy applications, it will be important to establish larger-scale process development systems to validate findings established at the smaller scale.

The ambr250® bioreactor platform has proven to be an effective scale-down model for clonal selection for CHO cell culture, resulting in comparable profiles of cell growth and protein production with 5 l and 1000 l bioreactor scales (Xu et al. 2017). This consistency of performance across the scales has resulted in expedited therapeutic development and reduced overall cost of development. Like the ambr15®, the ambr250® has the ability to individually control the pH and dissolved oxygen in individual bioreactors and benefit from automated liquid handling and sampling. However, the ambr250® has advantages over the ambr15® including the ability to control temperature individually in each bioreactor, the addition of four displacement pumps which can be used for base, acid, antifoam or feed addition, and the ability to individually control the agitation speed in each stirred-tank bioreactor. Moreover, as the volume is larger than that of the ambr15® (15 ml vs. 250 ml), this increase in scale provides the opportunity to generate sufficient material for downstream process development applications and material for analytical development.

For mammalian culture such as CHO clonal selection, the ambr250® uses a ‘mammalian’ vessel which comprises of two 20 mm pitched-blade impellers and four baffles; we refer to this vessel as the ‘original baffled’ vessel. In addition to CHO culture, the system has also been effectively demonstrated for other recombinant protein expression systems including *Escherichia coli* and *Pichia pastoris* where a ‘microbial’ vessel is used; this comprises of a two 20 mm Rushton turbine impellers (Bareither et al. 2013, 2015). The original mammalian ambr250® vessel however, the platform has not been demonstrated for cell and gene therapy applications such as hMSCs and T-cells. A primary reason for this omission is that initial studies undertaken by the technology manufacturer and others including ourselves have demonstrated that the currently available stirred-tank bioreactor vessel for mammalian cell culture in the ambr250® is sub-optimal for cultures which require microcarriers (e.g. adherent hMSCs) or activating beads (e.g. T-cell Dynabeads®) (Bareither et al. 2015; Costariol et al. 2019). The current vessel for mammalian cell culture comprises of a vessel with four baffles and a two, 20 mm pitch-blade impellers. Whilst this configuration has proven effective for CHO cultures amongst other free suspension mammalian cells, this vessel and impeller configuration does not appear to provide such satisfactory results for microcarrier cultures (e.g. adherent stem cells), or cultures where bead suspension is required (e.g. human T-cells). Specifically, its use has resulted in poor cell growth for adherent cell cultures and that involving Dynabeads due to the inability to effectively suspend the microcarriers/particles. The purpose of this work is to design and develop a new vessel which can improve and support the culture of cells which require effective suspension of beads for cell proliferation. Initial studies will focus on the engineering and physical characterization of the vessel and impeller geometries, followed by validation studies through CFD simulation and experimental studies with human T-cells with activating Dynabeads® and hMSC-microcarrier cultures.

## Materials and methods

### Power input, P and power number, Po

**Fig. 1:**
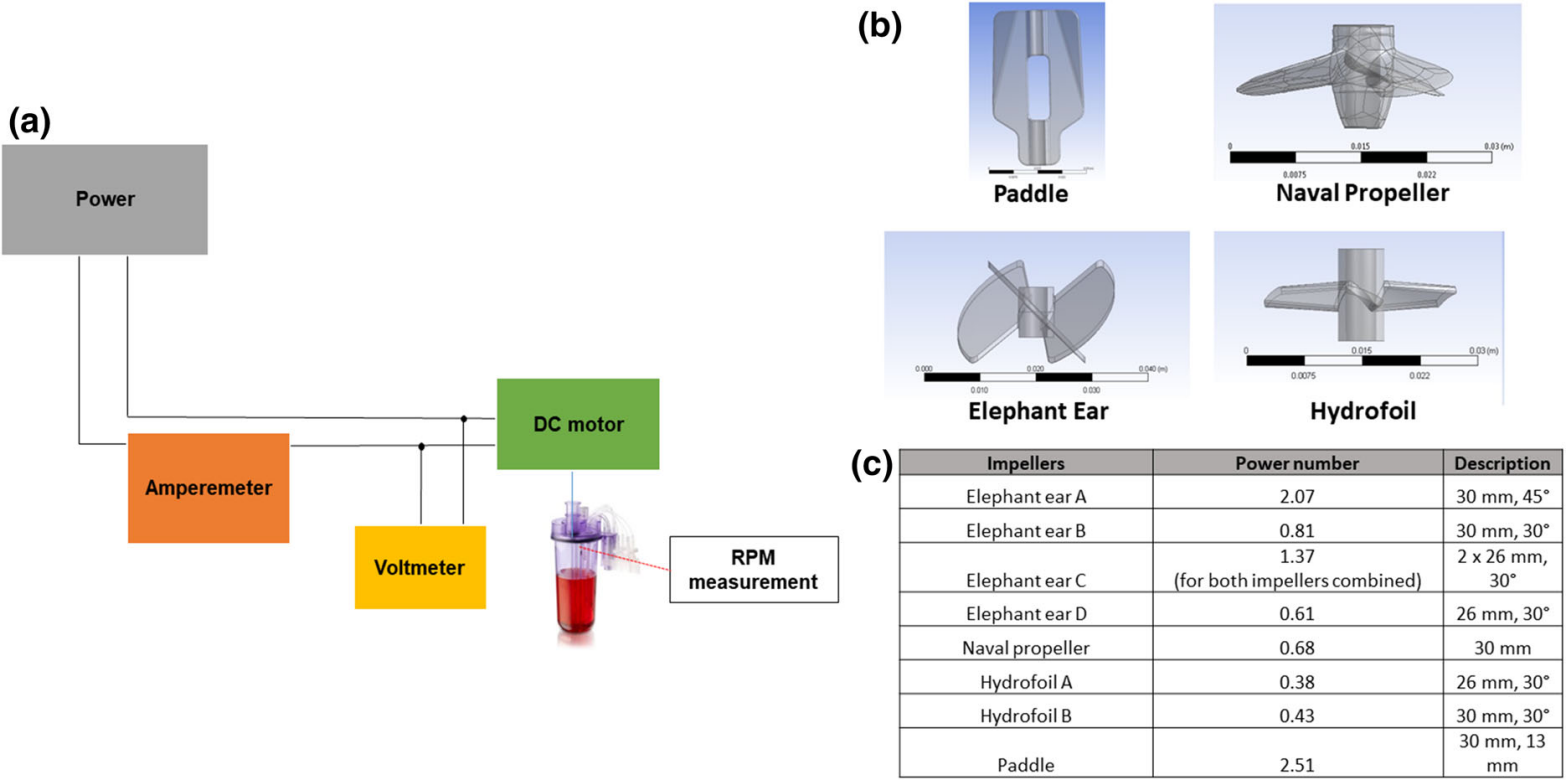
**a** A schematic of the experimental test rig for establishing the power number of each impeller, **b** a CAD image of the impeller types investigated including the paddle impeller (top left), the naval propeller (top right), the elephant ear impeller (bottom left) and the hydrofoil impeller (bottom right) and **c** properties of each of the impellers investigated including the power number measured and a brief description of the geometry

The power input (W) into the simulating medium was measured using an electric technique previously established for that purpose with the ambr®15 (Nienow at al., 2013) where the accuracy of the technique was also shown. In summary, the measurements were undertaken in a bespoke unbaffled ambr 250 bioreactor vessel with different impellers. The bespoke system has an identical geometry to that of a bioreactor used in the workstation, but with a DC motor that allows a wider stirring speed range. A schematic representation of the system can be found in Fig. 1a. The power demand was measured from the DC motor (RE-max 17 series, Maxon, Switzerland) directly coupled with the impeller. A range of impellers were used, the geometries and characteristics of which are provided in Fig. 1 and the measurement were undertaken with a fill volume of 200 ml. The base of the impeller drive shaft was modified to minimise the resistance losses and so the error of every measurement. The power was measured using a multimeter (Fluke 179, RS Components, UK) and checked against manufacturer’s specifications. Stirrer speeds from ~ 40 to ~ 2000 rpm could be obtained by varying the applied voltage, giving higher speeds than those employed when using the ambr250® as a bioreactor. These higher speeds were used in order to minimise the power drawn in overcoming friction compared to that required to drive the impeller thereby maximising the accuracy of the power input actually into the medium. At each point, the speed was optically measured with a manual laser tachometer (Wingoneer DT-2234C+) pointed to a reflecting tape placed on the impeller shaft. For each speed, the voltage and the current to the DC motor using the digital multimeter were measured in water with the impeller(s) of interest in place and in air without (to determine the frictional losses).

The total power input into the fluid, P (W), which drives all the mixing processes in a bioreactor (Nienow 2010) can then be calculated as:

1 $$P={P}_{M}-{P}_{R}-F,$$

where ${P}_{M}$ is the power to the motor (W), ${P}_{R}$ is the resistance due to electrical resistance and F is the frictional loss. The power loss for electrical resistance can be obtained from:

2 $${P}_{R}={I}^{2}R ,$$

where I is the current going to the motor (A) ant R is the resistance of the motor (Ω). If the vessel is empty (no water), we can assume that the resistance of the air is negligible (*P *= 0). Therefore, this allows F to be calculated.

The Power number could then be calculated using the following relationship.

3 $$Po=\frac{P}{\rho {N}^{3}{D}^{5}} ,$$

where P is the power input into the fluid from the impeller (W), $\rho$ is the fluid density (kg/m^3^), N is the impeller speed (rps) and D is the impeller diameter (m). The Power number is dependent on the Reynolds number, Re, of the system where for a stirred vessel it is defined as:

4 $$Re=\frac{\rho N{D}^{2}}{\mu } ,$$

where µ is the dynamic viscosity (Pa s). For bioreactors in which animal cells whether in free suspension or on microcarriers are grown, the viscosity of the media is close to water and the flow is turbulent (Re ≥ ∼ 2 × 10^4^) or nearly so, whatever the scale of the bioreactor and Po is constant, independent of Re. P_0_ could be determinate for each speed and thus the mean specific energy dissipation rate or specific power input, P/V (W/m^3^), from:

5 $$\frac{P}{V}=\frac{Po\rho {N}^{3}{D}^{5}}{V} .$$

Since to date, the oxygen demand for microcarrier culture can usually be met without sparging, the aerated Power number cases will not be considered here. In fact, even in free suspension culture, the sparge rate is generally insufficient to have an impact on power number (Nienow 2010).

### Just‐suspended (N_JS_) speed determination

With microcarriers added to the system, it is important to determine the just-suspended speed (N_JS_) for a given concentration of microcarriers. The N_JS_ is the speed at which the microcarriers are just completely suspended by the impeller (Nienow et al. 2016a), and was first used as the operating parameter for MSC culture in 2011 (Hewitt et al. 2011). Cytodex-1 (GE) is widely used in industry and was the microcarrier type first selected for this type of study (Rafiq et al. 2016; Schop 2010) though many others have been used too (Rafiq et al. 2016). The Cytodex-1 beads used were initially hydrated in PBS (Thermo Fisher Scientific, Loughborough, UK) for at least 3 h, washed with water and then added to the vessel at the appropriate concentration. As Cytodex-1 has transparent properties, a blue dye was used to make the particles visible.

The concentration of the Cytodex-1 varied between 1 and 5 g/l. The minimum suspension speed was determined by naked eye visual observation of the bottom part of the vessel, as previously described in Nienow et al. (2016b). Upon addition of the microcarriers, the vessel was not agitated to allow the microcarriers to settle on the bottom of the vessel. After a period of ~ 5 min, the impeller speed was sequentially increased to a value where all the microcarriers were completely suspended, based on the 1–2 s criterion (Zwietering 1958).

### CFD simulations

A computational fluid dynamics analysis was also performed using the commercial CFD solver Fluent (Ansys, Pennsylvania, United States) to provide a description of the vessel flow patterns and local liquid velocities. The simulation was undertaken for the ambr250 bioreactor vessel with different configurations of baffles/impellers. CAD drawings generated in Fluent of the impellers investigated for this study can be found in Fig. 1b. The liquid density was set as the water density (998 kg/m^3^) and viscosity (1 mPa s). The design was made available by Sartorius and directly imported and meshed. This design included the presence of the pH probe, the air sparger, the temperature probe and the vessel, impeller, shaft and walls (with or without baffles). The area around the impellers is defined as a moving reference frame (MRF) and represents the volume of fluid that rotates, driven by the agitator. The outer mesh is instead stationary with respect the outside vessel wall. All solid surfaces have a no-slip boundary conditions. The simulation is a steady state simulation, with residuals normalised at 10^−3^, with approximately 500 iterations. A single-phase simulation using the standard turbulent k-ε model was established for all CFD studies.

### T-cell isolation and expansion studies

Fresh peripheral blood mononuclear cells (PBMCs) from three healthy human donors were purchased from Cambridge Bioscience (UK) and T-cells were isolated as described in our previous paper (Costariol et al. 2019). The culture medium used in this study was Roswell Park Memorial Institute (RPMI) 1640 medium (Gibco® Thermo Fisher Scientific, Loughborough, UK) supplemented with 10% FBS, 2 mM l-glutamine (Thermo Fisher Scientific, Loughborough, UK), and 30 IU/ml interleukin-2 (IL-2; Milteny Biotech Ltd., UK). The cells were thawed in complete RPMI medium and activated using a 1:1 ratio of cell to Dynabeads® (Thermo Fisher Scientific, Loughborough, UK) and seeded in a T175 Nunc™ non-treated flask (Thermo Fisher Scientific, Loughborough, UK) at 37 °C and 5% CO_2_ in a humidified incubator.

The ambr250® bioreactor cultures involved using the appropriate impeller generally operated in the down-pumping mode which requires less specific power to achieve N_JS_ than up-pumping (Ibrahim and Nienow 2004) and is generally recommended (Nienow et al. 2016a, b). The ambr® 250 vessels were loaded and connected to the control system and 80 ml of RPMI 1640 with 10% FBS and 2 mM l-glutamine were placed in each vessel overnight to precondition the pH probe. The seeding procedure and feeding strategy is the same as that reported in our previous study (Costariol et al. 2019), with an initial seeding density of 0.5 × 10^6^ cells/ml in complete RPMI medium and medium additions on day 3 (100 ml), and day 4 (50 ml). The medium exchange on day 5 was performed by removing 100 ml of cell suspension from the bioreactor and centrifuging at 350×g for 10 min, resuspended in 100 ml complete RPMI medium and then added back to the ambr® 250 vessel. The medium addition/exchange strategy in the T-flasks (static control) resembled that of the ambr® 250 bioreactors. The agitator speed was set to 100 rpm.

### hMSC-microcarrier culture

Human mesenchymal stem cells (hMSC, RoosterBio) were cultured on Plastic microcarriers (Sartorius, Ann Arbor, United States) to investigate growth in the ambr® 250 system. The microcarrier and culture conditions selected were based on previous studies (Rafiq et al. 2013, 2016, 2018). In brief, 100 ml of hMSC culture media (PRIME-XV®, Irvine Scientific, California, United States) was added to the either the original baffled vessel or the newly developed unbaffled elephant ear impeller vessel (N = 2) and left in the vessel overnight to equilibrate the pH probe. The microcarriers were added to provide an initial surface area of 10 cm^2^/ml in the ambr vessels, and left in media for 2 h before inoculation. The hMSC were cultured in T-flasks, inoculated into the ambr250 bioreactors at 6000 cell/ml. During the first 2 h of the process, the stirring was intermittent between 0 and 100 rpm with a period of 10 min. On day 3 of the culture, 100 ml of hMSC culture media was added to each bioreactor, and a 50% media exchange was performed on days 5, 7, and 9, with an additional 2.5 cm^2^/ml of microcarriers added on days 5 and 9. The temperature was controlled at 37 °C. The impeller speed was initially fixed at 100 rpm, and increased in steps to 150 throughout the culture. The cells were harvested on day 10 with the same harvesting protocol used as described in Nienow et al. (2014). After harvesting on day 10, the cells were seeded in a 6 well plate and differentiated toward the adipogenic, chondrogenic and osteogenic lineages. This differentiation was achieved by culturing the cells in the respective differentiation medium for three weeks (Thermo Fisher Scientific, Loughborough, UK) and completed in line with the manufacturer’s instructions prior to staining to determine differentiation capability. Culture medium samples were analysed for glucose and lactate concentrations using the CuBiAn HT270 bioanalyser (4BioCell GmbH, Germany).

### Statistical analysis

Data analysis was performed using GraphPad Prism 7 software (GraphPad, La Jolla). Results are represented as mean ± SD. A one-way analysis of variance (ANOVA) test was used and values were considered statistically significant when probability (p) values were equal or below 0.05(*) or 0.01(**).

## Results and discussion

### Vessel and impeller modifications

#### Initial observations

The use of stirred-tank bioreactors for many cell and gene therapy applications requires the suspension of microcarriers or beads to facilitate cell growth. For anchorage-dependent cells such as hMSCs, the microcarriers provide a surface on which the cells adhere and proliferate. For suspension cell types, such as human T-cells, activation is commonly achieved through the use of magnetic Dynabeads®. T-cell activation is critical to support the function of T-cells and involves an intra-cellular signalling cascade that ultimately results in proliferation, effector function, or death, depending on the intensity of the activation (Panagopoulou and Rafiq 2019). The original vessel developed for the ambr250® was designed for free suspension cell cultures and has proved very effective (Xu et al. 2017). However, in work requiring the suspension of Dynabeads®, the initial performance was poor (Costariol et al. 2019). However, in work requiring the suspension of microcarriers or particles, the initial performance was poor as highlighted later in this study.

#### Particle suspension

Visual observations of the vessel during MSC culture identified that the microcarriers were poorly suspended. It was decided that to improve the growth of these cells, good suspension of the associated particles was essential and design improvements were therefore required. The observation of Dynabead® suspension is difficult because they are so small (Costariol et al. 2019) but it was suspected that poor suspension might be the cause of the poor culture performance in that case even though the agitation speed utilised was initially considered adequate.

One notable observation was the fact that microcarrier suspension was being impeded by the four baffles in the vessel, particularly collecting in the vortex behind them where they attach to the base of the bioreactor. In addition, sufficient Dynabeads® collected there too so that by careful observation they too could be seen. Previous studies have shown that unbaffled vessels and removal of probes which may impede the flow of beads are desirable for microcarrier or suspended bead culture due to this issue (Lundgren and Bluml 1998; Rafiq et al. 2017). The first design decision, therefore, was to remove the baffles from the original ambr250 vessel.

In addition to the removal of the baffles, it was recognised that improved bead suspension should be achieved through changes to the impeller geometry. The original ambr250 vessel has two, 20 mm pitched-blade impellers. Different impeller geometries and vessel configurations were investigated to identify which would be most appropriate for bead suspension.

#### Impact of changes on power number

**Fig. 2:**
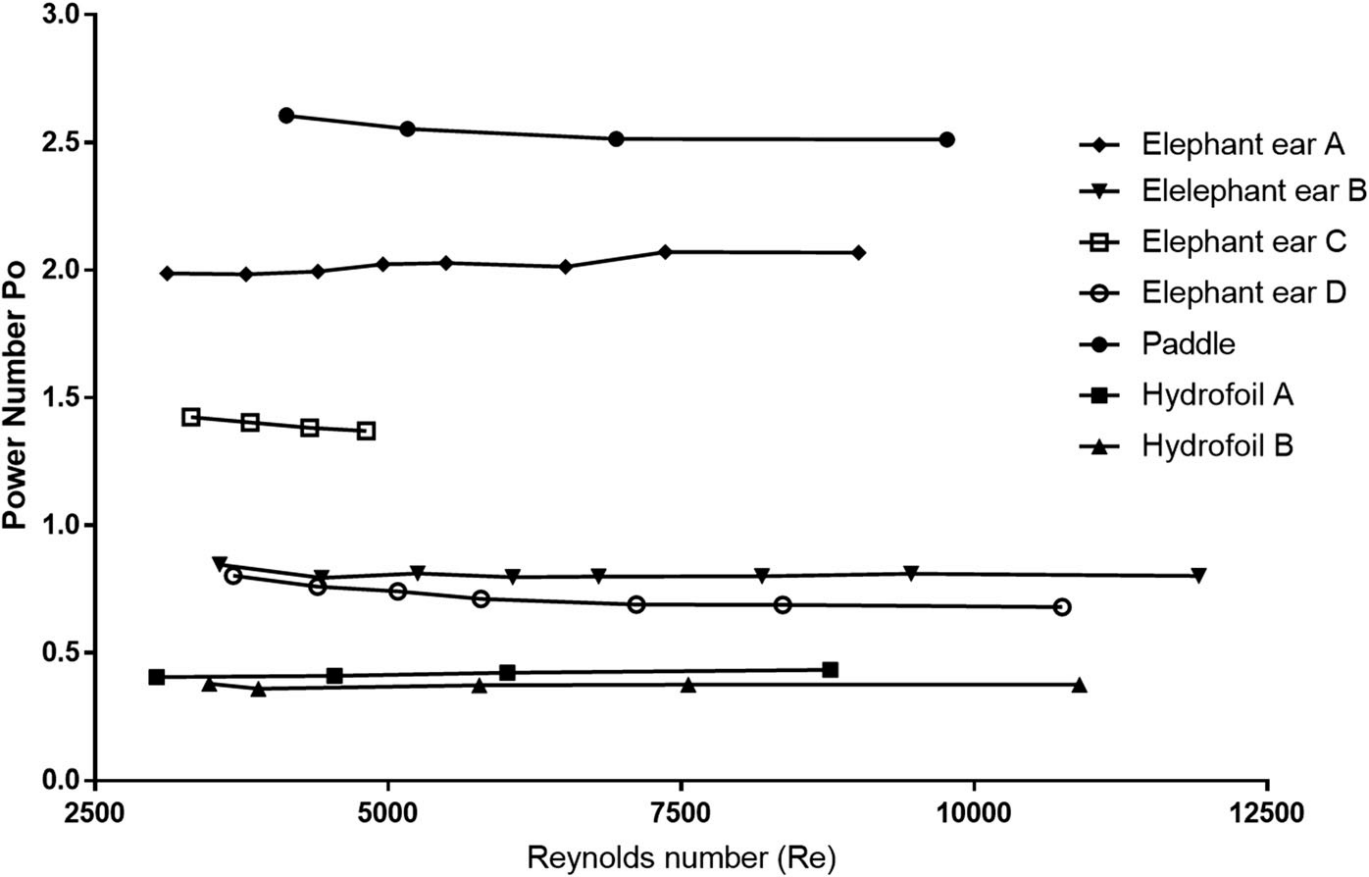
*Po* vs. *Re* values for each of the impellers investigated

Figure 1b provides a visual representation of the impeller configurations used and Fig. 1c provides details of the impeller geometries and associated average power numbers (*Po*) across the speed range. Figure 2 shows *Po* vs. *Re* for the different impeller geometries where the power imparted to the fluid in the vessel was sufficiently high compared to that required to overcome friction losses and electrical losses in the motor to give meaningful results. Even though the baffles had been removed, the simple circular swirling motion of the fluid found in vessels without baffles or inserts as in spinner flasks is disrupted by the presence of the essential probes required for monitoring and control. Hence, different flow patterns are seen and each geometry gives a different approximately constant power number as expected over this Reynolds number range (Ibrahim and Nienow 1995). Whilst the Po values at the lower Reynolds number shown in Fig. 2 were obtained at impeller speeds which are in the range of the measured NJS values, others are significantly higher than those that would be used during culture to maximise their accuracy, which is a typical strategy that has been used previously because Po is independent of Re in this Re range (Nienow et al. 2013).

As expected, a large paddle with blades at 90°, though selected to simulate a spinner flask, produced a radial flow because of the inserts and gave the highest power number, much higher than that actually found in spinner flasks (Hewitt et al. 2011). The elephant ear impellers have a *Po* ranging from 0.6 to 2.1, varying mainly due to the impeller blade angle (Tsui et al. 2006; Zhu et al. 2009). Also, as usual, the dual impellers (elephant ear C) give a combined Po approximately equal to twice that of the geometrically similar single impeller (elephant ear D) (Ibrahim and Nienow 1995). Finally, again as expected, the strongly axial flow impellers (propeller and the two hydrofoils) have an average *Po* of approximately 0.4, the lowest of all the impeller geometries. All these values are similar to those found in baffled vessels (Ibrahim and Nienow 1995).

#### Impact of impeller type on N_JS_ and associated specific power input, (P/V)_JS_

The minimum speed to just suspend the microcarriers (N_JS_) for all the impellers is shown in Table 1 along with the specific power input (P/V)_JS_ calculated from Eq. 5. The elephant ear A (EE/A) and paddle impeller gave the lowest N_JS_ (< 80 rpm), whilst for the other impellers, it ranged from 108 to 188 rpm. To some extent, the low values for N_JS_ for EE/A and the paddle are to be expected as the ability of an impeller to suspend particles depends on the mean specific power input and they have the highest power numbers. However, particle suspension also depends significantly on the flow pattern produced by the impeller and Table 1 also shows the paddle has the lowest specific power (W/m^3^). That finding is unusual because, in general, in baffled vessels, axial flow impellers suspend solids at much lower P/V than radial flow (Ibrahim and Nienow 1996). On the other hand, flat paddles in spinner flasks without baffles produce a strong swirling motion and suspend solids at very low (P/V). They also have Po of 1 or less in spinner flasks (Hewitt et al. 2011; Nienow et al. 2016a). The explanation for this low (P/V)_JS_ is probably that there is still a higher level of rotational flow in this bioreactor where baffling is only due to inserts into the vessel compared to the normal fully baffled case where swirling is largely prevented.

**Table 1:** Po, N_JS_ and (P/V)_JS_ for the different impeller configurations and vessel diameter, T = 60 mm

Impeller type	Po	N_JS_ (rpm)	N_JS_ (rps)	Impeller diameter (D, m)	Density (kg/m^3^)	Volume (l)	P/V_JS_ (W/m^3^)	P/V_JS_ rank order
Paddle	2.51	70	1.17	0.03	1000	0.2	4.84E−04	1
Elephant ear A	2.07	76	1.27	0.03	1000	0.2	5.11E−04	2
Elephant ear B	0.81	108	1.80	0.03	1000	0.2	5.74E−04	3
Elephant ear C (2 impellers)	1.37	146	2.43	0.026	1000	0.2	1.17E−03	6
Elephant ear D	0.61	171	2.85	0.026	1000	0.2	8.39E−04	5
Naval	0.68	146	2.43	0.03	1000	0.2	1.19E−03	7
Hydrofoil A	0.38	184	3.07	0.026	1000	0.2	6.51E−04	4
Hydrofoil B	0.43	188	3.13	0.03	1000	0.2	1.61E−03	8

Studies for N_JS_ were undertaken in water with a Reynolds number range of 3500 to 9400

The other N_JS_ values for shallower blade angle and smaller diameter EE impellers are typically higher as the Po decreases; and for the dual impeller, N_Js_ remains similar to that for the single impeller even though the Po is approximately doubled, indicating the importance of the flow pattern at the base of the bioreactor on particle suspension (Ibrahim and Nienow 1995, 2004). Finally, the hydrofoils and propeller even though they produce a strong axial flow which is important for efficient suspension, have the some of the highest values of N_JS_. One of the issues with axial flow impellers in baffled vessels is that as the impeller diameter, D, approaches 50% of the vessel diameter, T, the flow loses its strong axial motion and (P/V)_JS_ increases significantly. That might be the reason why for the propeller and larger hydrofoil B where D/T = 0.5, (P/V)_JS_ are the two highest of all the impellers tested whilst for hydrofoil A, (D/T = 0.43), (P/V)_JS_ is close to the values for the two most efficient EE impellers and significantly below the others.

The addition of cells seemed to increase N_JS,_ especially once the attached, cells began to cause bridging between microcarriers, thereby increasing its size. For example, in the unbaffled vessel with elephant ear impeller A, N_JS_ for Cytodex-1 without cells was about 80 rpm, whilst with cells, it increased to 105 rpm.

#### Fluid dynamic stress issues

For microcarrier cell culture applications, there are two primary stresses which may cause cellular damage; (1) fluid dynamic stress arising as a result of turbulence and (2) stresses associated with microcarrier–microcarrier impacts or microcarrier-impeller impacts. We have discussed this in detail previously (Nienow et al. 2014) and though rapid increases in the impact stresses with increasing speed have proved to be an effective way of detaching cells from microcarriers in the presence of detachment enzymes during harvest, there is no evidence to suggest that damage occurs either during that process or culture at N_JS_. On the other hand, it has also been found that the impact of fluid dynamic stress from turbulence can be effectively considered in relation to the size of the Kolmogorov scale in relation to the size of the biological entity (Nienow 2020). Typically, in brief, if the size of the biological entity is less than the Kolmogorov scale, λ_K_, the cell will not be damaged in stirred bioreactors. However, in the case of cells on microcarriers, it is not immediately obvious which of the cell size or the microcarrier size should be considered appropriate. In early work on cell culture on microcarriers, it was found that provided the Kolmogorov scale was > 2/3rd of the microcarrier size, the cells would not be damaged (Croughan et al. 1988). In our more recent work, the use of N_JS_ was proposed as the basis for microcarrier culture, and that was found to be effective from a 5 l Sartorius Stedim bioreactor down to the ambr®15 (Nienow et al. 2016b) and it has been adopted elsewhere (for example, Jossen et al. 2018). In the ambr®15, the Kolmogorov scale at N_JS_ was only about 25% of the size of the microcarriers (Rafiq et al. 2017) yet the culture was undertaken quite successfully.

So though there is considerable evidence that microcarrier based culture of stem cells can be successfully undertaken at N_JS_, it is clearly sensible to try to minimise the specific power required to achieve that condition as.

6 $$\lambda _{{\text{K}}} \propto ({\text{P}}/{\text{V}})_{{{\text{JS}}}}^{{ - 1/4}} .$$

and so the lower the value of (P/V)_JS,_ the greater the flexibility in operating conditions. For example, this extra flexibility could be required when wishing to increase agitator speed to enhance oxygen mass transfer as higher cell densities are achieved. That increase itself might arise when adding more microcarriers, which in itself increases N_JS_, to take advantage of bead-to-bead cell transfer (Rafiq et al. 2018). In addition, it was observed during this study that N_JS_ also increased as cells grew on the microcarriers especially when it caused microcarrrier clumping and in maintaining suspension clumping was itself reduced.

As a result of these considerations, the importance of minimising (P/V)_JS_ is very clear. The lowest is the paddle. However, radial flow impellers are very rarely if ever used at larger scales in stirred bioreactors for animal cell culture, whether free suspension or on microcarriers. Therefore, it was decided to proceed with the elephant ear impeller A for which (P/V)_JS_ was only slightly higher and is a common shape in this technology.

### CFD simulation of flow using elephant ear A impeller in the ambr250

**Fig. 3:**
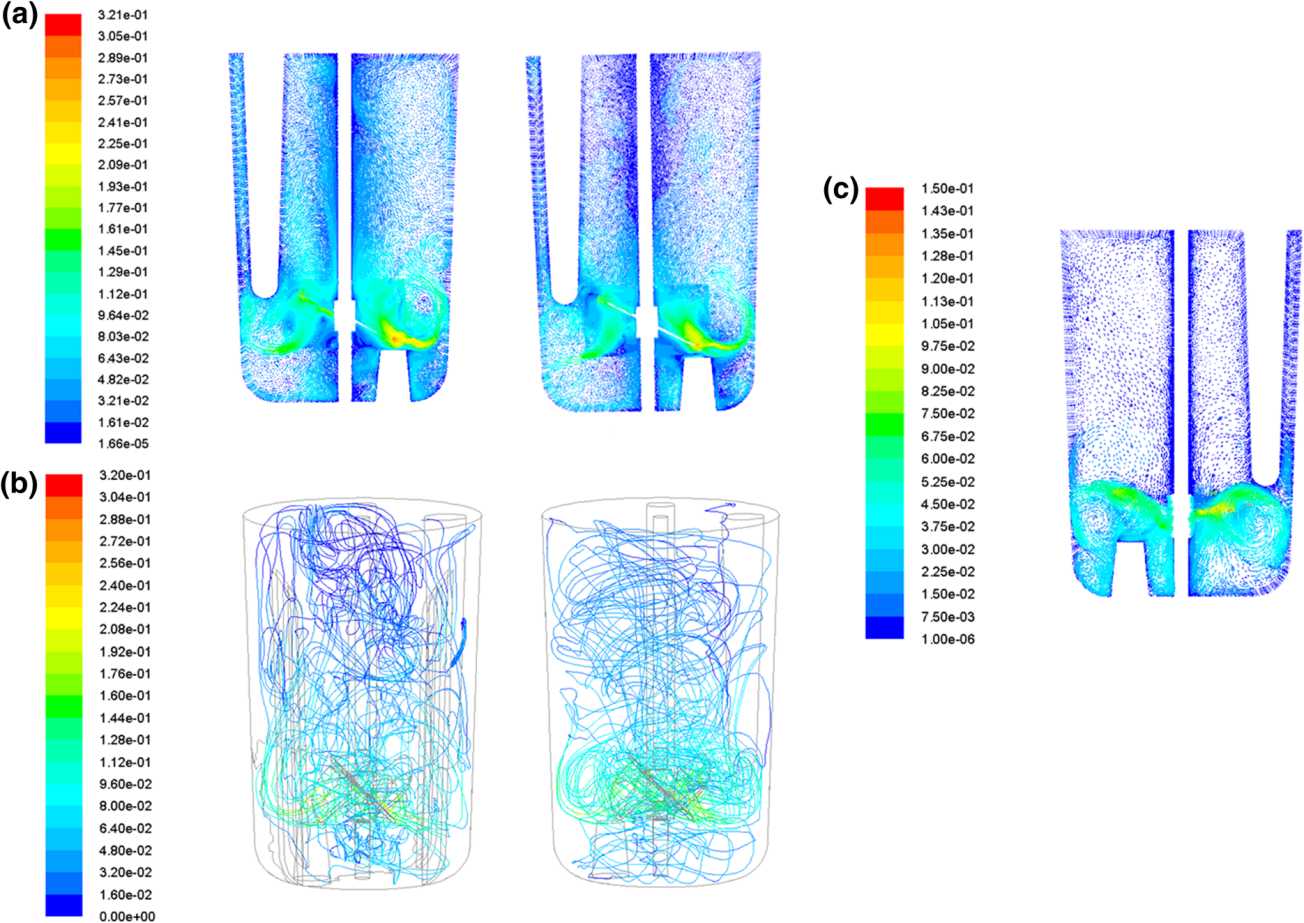
**a** CFD simulations for the baffled (left) and unbaffled (right) vessels employing the elephant ear A impeller, **b** the flow path simulations for the baffled (left) and unbaffled (right) vessels employing the elephant ear A impeller and **c** the CFD simulation for the unbaffled vessel employing a naval propeller impeller

Figure 3a shows the velocity vector maps and flow patterns for the elephant ear A impeller in both the baffled (left) and unbaffled (right) vessels at the same stirring speed (150 rpm, down pumping). The two flow patterns are similar, exhibiting a mixture of radial and axial flow, similar to that found with earlier experimental PIV-based studies in larger baffled vessels with such impellers (Zhu et al. 2009). This similarity in flow pattern between the baffled and unbaffled cases also implies that though the traditional baffles have been withdrawn to eliminate dead zones as discussed above, the presence of the various inserts at this small scale still gives sufficient baffling to give all the advantages that come from a baffled construction (Pogal and Kehn 2018) without the disadvantages with respect to particle suspension. This difference is reinforced by the fluid path line simulations (Fig. 3b) for the baffled (left) and unbaffled (right) vessels where it can be seen that in the volume below the impeller, there is a lot more motion in the unbaffled vessel, spreading out across the bottom of the vessel, compared with the baffled vessel where the flow below the impeller appears restricted to the bottom-centre of the vessel and hindered by the baffles. This region is critical for the suspension of microcarrier and beads in cultures requiring their use and the flows illustrated for the unbaffled vessel in Fig. 3a, b are highly effective at encouraging that condition. Figure 3a, b also show flow towards the bottom part of both vessels is somewhat impeded by the temperature uplift and the probes in the vessel. However, to ensure effective process monitoring and control, these aspects of the vessel design could not be changed. They also encourage the downward flow rather than just a simple swirling motion.

In contrast to the elephant ear A impeller, a CFD flow simulation was undertaken with a naval propeller in an unbaffled vessel (Fig. 3c) under similar conditions to the elephant ear A studies described above. Notably, the fluid flow is predominantly radial in nature, with the simulation suggesting that the naval propellor is unable to generate an effective axial flow regime, a finding that supports the findings in “Vessel and impeller modifications” section. Based on the findings from the impeller power and suspension studies and the CFD simulations, it was decided that the most promising vessel and impeller configuration for microcarrier/bead-based cultures was the elephant ear A impeller (30 mm diameter, 45° pitched-blade angle) in an unbaffled vessel. However, before finally selecting this impeller, it would be important to validate this with experimental studies using microcarriers/beads and cells.

### Primary human T-cell culture with activating Dynabeads®

**Fig. 4:**
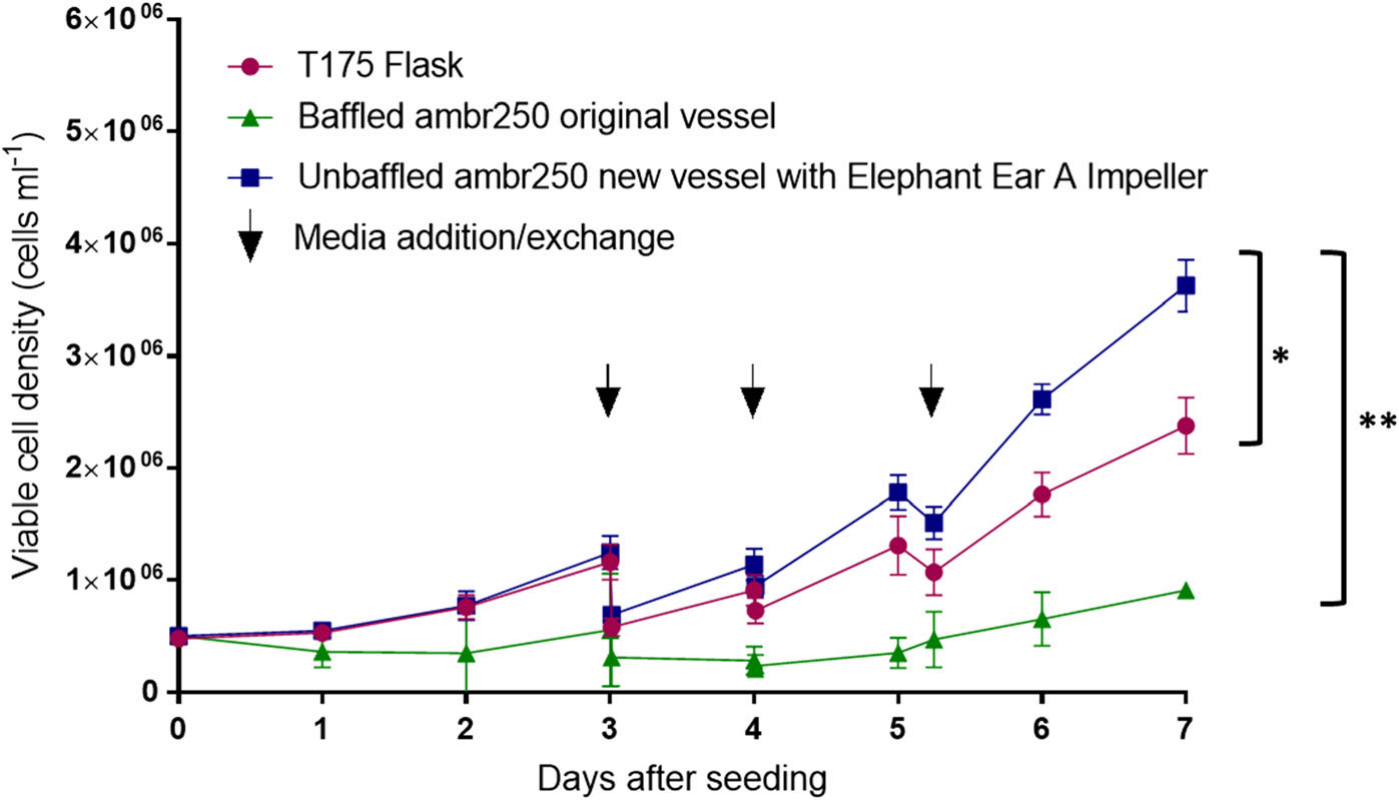
Human T-cell viable cell density in the T-flask, the original baffled ambr250 vessel and the unbaffled elephant ear A impeller vessel (n = 3). Data shown as mean ± SD. The black arrows indicate a medium addition (days 3 and 4) and exchange (day 5). Statistical difference (P) values were equal to or below 0.05(*) or 0.01(**)

The growth kinetics of primary human T-cells in static T-flasks, the original baffled ambr250 vessel with impeller EE/C and the newly designed unbaffled ambr250 vessel with impeller EE/A is shown in Fig. 4. It is notable that the unbaffled vessel with the impeller EE/A results in a significantly higher viable cell density by the end of the culture (4 × 10^6^ cells/ml) compared with the static T-flask culture (~ 2.75 × 10^6^ cells/ml) and the original baffled vessel with impeller EE/C (~ 1.0 × 10^6^ cells/ml). The reasons for this are discussed below.

In the baffled bioreactor with impeller EE/C, at the agitator speed employed of 100 rpm, beads could be seen collecting at the base of the baffles and although T-cells grow in free-suspension, they have to be activated by close contact between cells and beads as discussed in detail previously (Costariol et al. 2019). In summary, that study showed because T-cells are of almost neutral buoyancy, they become suspended at very low impeller speeds when the functionalised Dynabeads®, which though very small are relatively very dense, are not. Under these conditions, very poor contact occurs leading to even worse activation than in static T-flasks. On the other hand, with the removal of the baffles and the higher P/V as a result of the greater power number of EE/A, collections of beads are not seen and good contact occurs in suspension enabling T-cell growth and activation.

Overall, the performance is better than in static T-flasks showing that with the improved geometry of the bioreactor plus the use of the elephant ear impeller A, the fluid dynamic regime in a stirred bioreactor enhances the culture of T-cells compared to static conditions.

### Primary human mesenchymal stem cell microcarrier culture

**Fig. 5:**
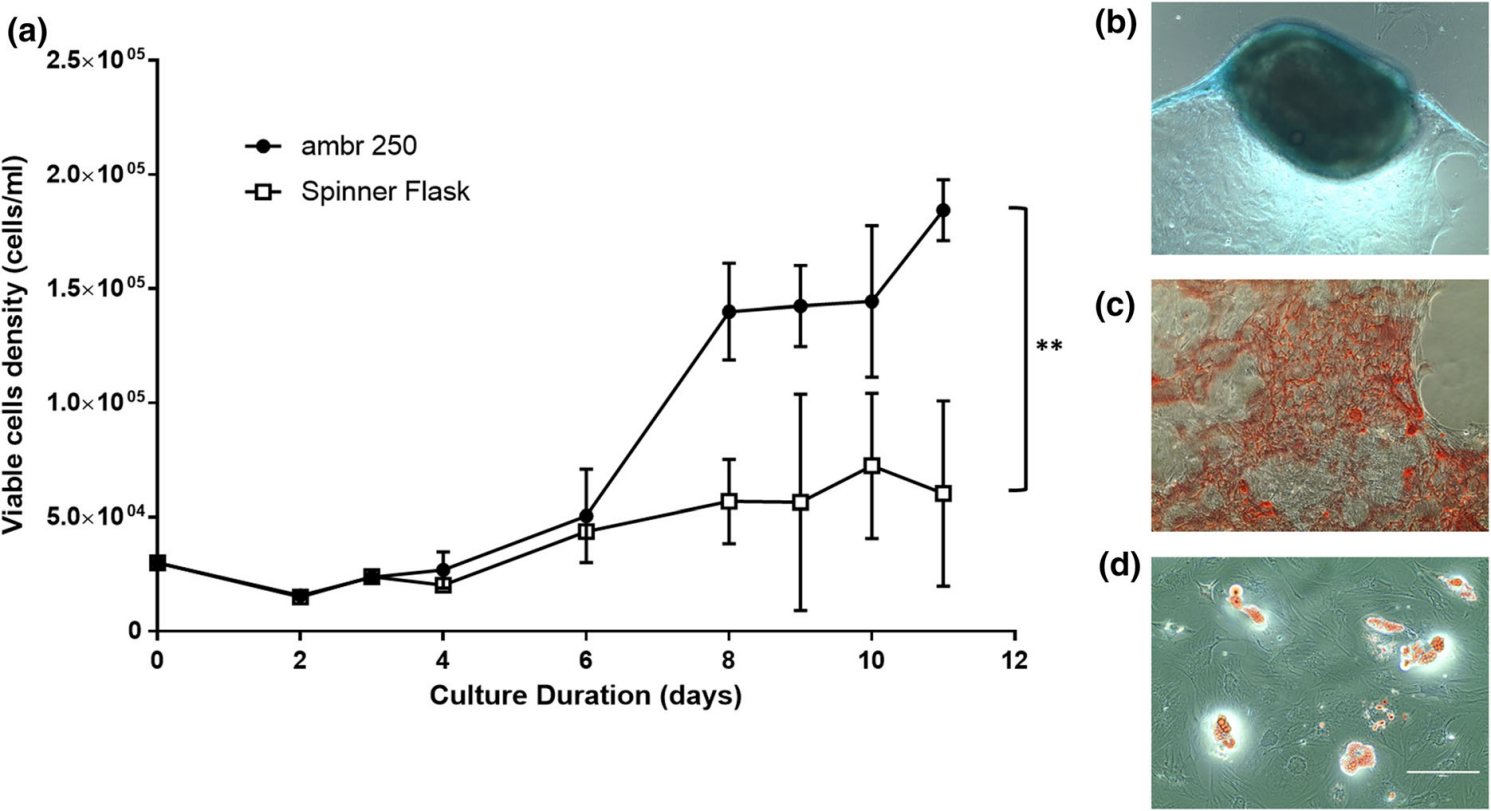
**a** Viable cell density for human mesenchymal stem cells on plastic microcarriers cultured in the new unbaffled ambr250 vessel and elephant ear A impeller and spinner flasks. Statistical difference (P) values were equal to or below 0.01(**). **b** Chondrogenic differentiation of harvested hMSCs stained with Alcian Blue. **c** Osteogenic differentiation of harvested hMSCs stained with Alizarin Red, and **d** adipogenic differentiation of harvested hMSCs stained with Oil Red O. Scale bar represents 100 µm

Having demonstrated the improved culture of primary human T-cells in the unbaffled vessel with elephant ear A impeller, the primary culture of human mesenchymal stem cells on microcarriers was also demonstrated and compared with magnetically-driven spinner flasks (Fig. 5a). A viable cell density of 1.83 × 10^5^ cells/ml was achieved with the unbaffled vessel by day 10 which was significantly higher than the maximum cell density of 6.0 × 10^4^ cells/ml achieved in the spinner flasks by day 9 (the corresponding day 10 density in the spinner flask was 4.7 × 10^4^ cells/ml). Identical conditions were employed in both systems, with both platforms being uncontrolled with respect to the dissolved oxygen concentration and pH to maintain parity between the systems. Cells cultured in the ambr250 vessel were harvested as described in Nienow et al. (2014) and were differentiated toward the chondrogenic (Fig. 5b), osteogenic (Fig. 5c) and adipogenic (Fig. 5d) lineages. This proof-of-principle study demonstrating the growth of hMSCs on microcarriers in the newly designed vessel shows significant promise and highlights the capability of the unbaffled ambr250 vessel for regenerative medicine and cell and gene therapy applications. Spinner flasks are commonly used for hMSC-microcarrier culture (Dos Santos et al. 2011a, b; Rafiq et al. 2013, 2016; Schop et al. 2010). However, with the improved performance of the newly designed unbaffled vessel with the elephant ear impeller, the additional benefits of automated liquid handling, improved process monitoring and control capability, and the high-throughput capacity of the platform (up to 24 bioreactors can be run simultaneously), the ambr250 becomes a key platform for cell and gene therapy process development.

## Conclusions

The research undertaken in this study demonstrated the design and development of a new bioreactor vessel and impeller for the ambr250® high-throughput, automated bioreactor platform, resulting in improved cell production for microcarrier and bead-based cultures. The new vessel was designed with a view to improve the suspension of microcarriers/beads given the inability of the original ambr250® to effectively suspend particles. The design and development of the new vessel was based upon both engineering and physical characterisation studies where a range of different impeller geometries were investigated and characterised with respect to the minimum speed at which they just suspended microcarriers (N_JS_) and their specific power at N_JS_ (P/V)_JS_. Of the various impeller geometries tested, the elephant ear A impeller (30 mm impeller diameter (D/T = 0.5) and with a 45˚ pitched-blade angle) was selected on the basis of it being the impeller requiring the least (P/V)_JS_. By considering the implications of cell damage from fluid dynamic stress via the Kolmogorov scale of turbulence, it was also shown that the low (P/V)_JS_ value gives the greatest operational flexibility when dealing with cell culture in the presence of particles.

CFD simulations were also undertaken to determine the fluid flow and help validate the physical characterisation studies. The CFD indicated that the unbaffled vessel would result in more vigorous motion at the bottom of the vessel, thereby encouraging suspension from the region where particles most easily settle. The CFD also confirmed that the presence of inserts, essential for monitoring and controlling the bioreactor performance, provided sufficient baffling that the flow patterns found in baffled bioreactors with different impeller shapes remained.

Finally, experimental studies with T-cells and hMSCs were used to demonstrate that the new vessel and impeller could support the culture of these important cell and gene therapy cell candidates. The new vessel resulted in significantly higher cell densities for T-cell Dynabead® cultures compared to the original ambr250® and static T-flask culture. The new vessel also demonstrated the ability to support hMSC microcarrier cultures and resulted in higher cell densities compared with spinner flask cultures.

This study has shown that new ambr250® platform gives significant improvement over the original vessel for cell and gene therapy applications involving beads and microcarriers and will support process development activity for cellular therapies. Of course, it is also suitable for free suspension culture too.

## Data Availability

All data is made available and presented in the manuscript.
